# Time Matters: Methane Inhalation Mitigates Mitochondrial and Organ Dysfunction in Advanced Experimental Sepsis

**DOI:** 10.3390/antiox14070814

**Published:** 2025-07-01

**Authors:** Levente Frigyes Gulácsi, Attila Rutai, László Juhász, Bálint László Czakó, Andrea Szabó, Mihály Boros, József Kaszaki, Marietta Zita Poles, Szabolcs Péter Tallósy

**Affiliations:** Institute of Surgical Research, Albert Szent-Györgyi Medical School, University of Szeged, 6720 Szeged, Hungary; gulacsilevente@gmail.com (L.F.G.); rutai.attila@med.u-szeged.hu (A.R.); juhasz.laszlo.1@med.u-szeged.hu (L.J.); czakobalint61522@gmail.com (B.L.C.); szabo.andrea.exp@med.u-szeged.hu (A.S.); boros.mihaly@med.u-szeged.hu (M.B.); kaszaki.jozsef@med.u-szeged.hu (J.K.); poles.marietta.zita@med.u-szeged.hu (M.Z.P.)

**Keywords:** fecal peritonitis, methane post-treatment, oxidative phosphorylation, kidney, cerebellum

## Abstract

This study aimed to characterize the time-dependent effects of methane (CH_4_) inhalation, initiated at defined intervals following sepsis onset, on organ function, systemic oxygen utilization, and mitochondrial respiration in a rodent model. Adult rats were subjected to abdominal sepsis or sham operation. Septic animals were assigned to groups receiving 2.2% CH_4_ in normoxic air at specific post-insult phases (early: 3–6 h; intermediate: 16–19 h; late: 19–22 h), while a control group remained untreated. At 24 h, organ function was evaluated using a Rat-Specific Organ Failure Assessment (ROFA) score, along with measurements of plasma myeloperoxidase (MPO) activity, Complex I–II-linked oxidative phosphorylation in renal and cerebellar tissues, systemic oxygen extraction, and global tissue perfusion (pCO_2_-gap). Sepsis induced significant organ dysfunction, impaired hemodynamics, reduced oxygen utilization, and decreased mitochondrial respiration. CH_4_ inhalation improved survival when administered early, restored cerebellar mitochondrial respiration during the intermediate phase, and in the late phase reduced ROFA scores and MPO levels, while attenuating mitochondrial dysfunction in renal and cerebellar tissues. All CH_4_-treated groups demonstrated improved renal function and enhanced tissue oxygenation. Targeted CH_4_ inhalation during sepsis confers protective effects by preserving mitochondrial function, reducing inflammation, and improving oxygen dynamics, suggesting promising therapeutic potential.

## 1. Introduction

Sepsis is a life-threatening syndrome caused by a dysregulated host response to infection, causing organ failures [1]. As sepsis progresses, an oxygen delivery (DO_2_)–consumption (VO_2_) mismatch transpires, with cellular deficits in oxygen extraction (ExO_2_) limiting mitochondrial oxygen availability [2]. Mitochondrial dysfunction is crucial to pathology, impairing ATP production, shifting metabolism to anaerobic pathways, and generating reactive oxygen species (ROS), all contributing to tissue damage and organ failure [3,4]. Cardiovascular dysfunction is an early clinical complication, resulting in central nervous system abnormalities in up to 70% of patients, as measured using the Sequential Organ Failure Assessment (SOFA) score [5,6]. Another severe complication that is associated with significantly higher mortality rates is sepsis-induced acute kidney injury (AKI), driven by renal hypoperfusion and inflammation [7,8]. Despite progress in critical care, novel treatment strategies targeting sepsis are still needed [9,10]. As conventional respiratory and circulatory support frequently fails to address sepsis-induced organ alterations, drug delivery efficacy remains a key determinant of therapeutic success [11,12]. Considering that subcellular, cellular, and intercellular membrane components are central to sepsis pathophysiology, agents that can traverse various biological barriers are particularly well-suited for this application. Although the anti-inflammatory, antipyroptotic, and antiapoptotic effects of methane (CH_4_) have been well-characterized [13,14], as well as several components of the signaling mechanisms, including its ability to suppress cytokines and inhibit NF-κB activation [15,16,17], its role in sepsis-induced mitochondrial dysfunction and organ failure remains unexplored. This is the first study to investigate the therapeutic role of inhaled CH_4_ in experimental sepsis, focusing on mitochondrial preservation and organ function. Notably, inhaled CH_4_ can be directly delivered to the lungs, and CH_4_ (2.2%) administered in a normoxic air mixture has been shown to preserve renal function in preclinical models of extracorporeal perfusion and veno–venous membrane oxygenation [18]. Based on previous findings, we hypothesized that inhaled CH_4_, a biologically active gas that can cross cellular and subcellular membranes [13], could enhance mitochondrial function and concurrently alleviate organ dysfunction in experimental sepsis. We aimed to evaluate the temporal dynamics of mitochondrial respiratory impairment and organ dysfunction over a 24-h period in a polymicrobial rat model of sepsis, both with and without CH_4_ treatment [19]. Furthermore, we sought to determine the optimal therapeutic window for CH_4_ administration. Septic animals were randomized into untreated and CH_4_-treated groups, with identical treatment durations applied at distinct stages of sepsis progression (3–6, 16–19, and 19–22 h following sepsis induction).

## 2. Materials and Methods

Experiments were conducted on 11–12 weeks old male Sprague Dawley rats (*n* = 44; 380 ± 30 g). Animals were housed under controlled conditions, appropriate temperature (21–23 °C), and humidity (45–55%) in plastic cages with a 12/12 h dark/light cycle and access to standard rodent food and water ad libitum. These conditions were maintained throughout their lifetime to ensure consistent living conditions. The study was conducted in a randomized, double-blind manner. Neither the individual performing the experimental procedures nor the investigator analyzing the samples was aware of the groups of animal allocations. This approach allowed both the execution of the experiments and the analysis of the data to be performed without bias. All procedures followed the National Institutes of Health guidelines and EU Directive on the care and use of laboratory animals, with the study protocol approved by the local Institutional Review Board and the National Scientific Ethical Committee for Animal Experimentation in Hungary (license V./2884/2022, approved: 14 November 2022). The study design and data presentation followed the Minimum Quality Threshold in Preclinical Sepsis Studies (MQTiPSS) recommendations [20] and ARRIVE guidelines (https://arriveguidelines.org/).

### 2.1. Experimental Protocol

Sample size estimation was performed, assuming approximately 20% mortality after 24 h. If the presumed true hazard ratio of septic subjects relative to controls is 0.2 with a power of 1-β = 0.8 and the Type I error probability is α = 0.05, the inclusion of 9 animal/group was recommended.

**Figure 1:**
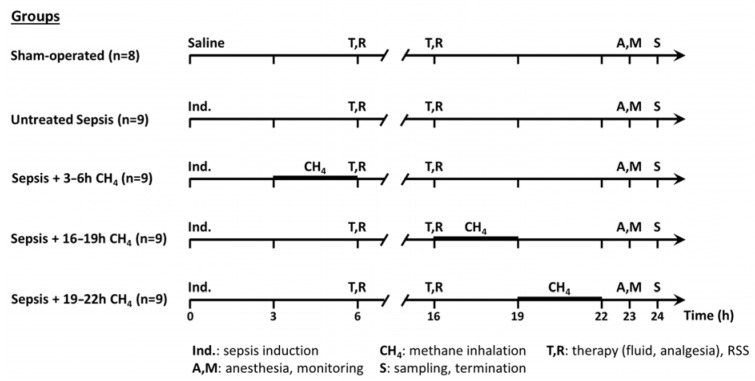
Scheme of the experimental protocol (groups, interventions, and assessments).

The experimental animals were randomly assigned to a sham-operated group (*n* = 8) or septic groups (n∑ = 36) [19]. The septic groups were intraperitoneally (i.p.) injected fecal inoculum (t = 0 h) to induce peritonitis, ensuring the rapid onset of polymicrobial sepsis. Previously, fresh feces were randomly collected from healthy rats (*n* = 5). Four grams of fecal material were mixed with 36 mL of saline and incubated for 6 h at 37 °C. The resulting suspension was filtered, and a 5 mL/kg inoculum was i.p. administered using a 21-G needle. The septic groups were further categorized into an untreated group (*n* = 9) and three CH_4_-treated septic groups (see below). The sham-operated group received an equivalent volume of sterile physiological saline (Figure 1).

Sepsis progression was monitored using a modified rat-specific well-being-related sickness (RSS) score system [19] at baseline and at 6 and 16 h post-induction. At the time of the RSS assessments, the animals received 10 mL/kg crystalloid solution subcutaneously (sc) (Ringerfundin, B. Braun, Hungary) to avoid dehydration and 15 µg/kg buprenorphine sc (Bupaq, Merck, USA) to maintain analgesia according to the MQTiPSS recommendations [20]. At 23 h post-induction, the animals were anesthetized with a mixture of ketamine (45.7 mg/kg; Medicus Partner Ltd., Biatorbágy Hungary) and xylazine (9.12 mg/kg; Produlab Pharma, Raamsdonksveer, The Netherlands) i.p. Subsequently, the animals were positioned supine on a heated operating table to maintain normothermia (37 °C). To secure patent airway, tracheostomy was performed. A PE50 cannula was inserted into the right external jugular vein for continuous anesthesia (ketamine 12 mg/kg/h, xylazine 2.4 mg/kg/h and diazepam 0.576 mg/kg/h; Richter Pharma, Hungary), fluid replacement (10 mL/kg/h; Ringerfundin, B. Braun, Budapest, Hungary), and venous blood sampling. Moreover, the left carotid artery was cannulated for continuous heart rate and mean arterial pressure (MAP) monitoring (SPEL Advanced Cardiosys 1.4, Experimetria Ltd., Budapest, Hungary), as well as for arterial blood gas and lactate analysis. At 24 h post-induction, median laparotomy was performed. Venous blood was collected from the inferior vena cava in tubes containing EDTA (for myeloperoxidase [MPO] level analysis). The left kidney was removed, and the animals were subsequently euthanized by rapid decapitation under deep anesthesia. The skull was opened along the sagittal suture, the cerebellum was separated from the adjacent brain tissue, and cerebellar and renal tissue samples were obtained for biochemical and mitochondrial analyses.

### 2.2. Methane Treatment

For CH_4_ treatment, conscious animals were placed in a sealed chamber with a pressure-compensating valve at 3-h intervals at their respective group timepoints (t = 3–6, 16–19, or 19–22 h post-induction). The chamber was presaturated with normoxic artificial air containing 2.2% CH_4_, 21% O_2_, and 76.8% N_2_. A continuous flow rate of 250 mL/min was maintained and monitored using a rotameter. Bedding, food, and water were provided throughout the inhalation period.

### 2.3. Organ Failure Assessment

The SOFA scoring system, originally developed for humans, was previously adapted by our group to incorporate rodent-specific parameters [21]. Respiratory function was assessed using the ratio of arterial oxygen partial pressure (PaO_2_) to fractional inspired oxygen (FiO_2_, set at 0.21 for room air). MAP was used for evaluating cardiovascular status. Blood lactate levels indicated metabolic imbalance due to anaerobic metabolism. Kidney injury severity was assessed on the basis of plasma urea concentrations; plasma alanine aminotransferase (ALT) levels, measured using a Roche/Hitachi 917 analyzer (F. Hoffmann–La Roche AG, Basel, Switzerland), were used for liver dysfunction evaluation. Animals with ROFA scores of >2 were classified as septic. The resulting Rat-Specific Organ Failure Assessment (ROFA) scores were used for evaluating multiple organ dysfunction in animals, with each component assigned a score of 0–4 on the basis of predefined thresholds (Supplementary Material, Supplementary Table S1).

### 2.4. Measurements of the Oxygen Dynamics

Using a Cobas b 123 blood gas analyzer (Roche Ltd., Basel, Switzerland), blood gas variables were measured from heparinized arterial and venous samples. Simplified ExO_2_ was calculated using the formula (SaO_2_ − SvO_2_)/SaO_2_, based on arterial (SaO_2_) and venous oxygen saturation (SvO_2_). Tissue perfusion quality was assessed by determining the pCO_2_ gap, defined as the difference between venous and arterial CO_2_ partial pressures (pCO_2_ gap = PvCO_2_ − PaCO_2_).

### 2.5. Mitochondrial Respiration Assessment

Mitochondrial oxygen consumption linked to mitochondrial Complexes I and II (C-I and C-II) was evaluated in cerebellar and kidney homogenate samples using high-resolution respirometry (Oxygraph-2k; Oroboros Instruments, Innsbruck, Austria) [21]. Briefly, LEAK respirations were evaluated after oxidation of the following complex specific substrates: 10 mM glutamate, 2 mM malate (C-I–linked respiration; LEAKGM), or 10 mM succinate (C-II–linked respiration; LEAKs). Before adding succinate, C-I–linked respiration was inhibited with rotenone (0.5 μM). Maximal capacities of oxidative phosphorylation (OXPHOS I and II) were achieved by saturating the ADP concentration (2.5 mM). Exogenous cytochrome c (CytC; 10 μM) was used after adding ADP to assess the outer membrane integrity. ATP synthase was inhibited by oligomycin (2.5 μM) to evaluate LEAK respiration in a nonphosphorylating state (LEAKOmy) of mitochondrial respiration. Electron transport-independent respiration (or residual mitochondrial oxygen consumption, ROX) was determined following C-III inhibition with antimycin A (2.5 μM). All measurements were performed under continuous stirring (750 rpm) at 37 °C in a 2 mL Mir05 respiration buffer. Mitochondrial oxygen consumption was expressed in pmol/s/mL. All respiratory substrates and inhibitors were purchased from Sigma–Aldrich (St. Louis, MO, USA). The DatLab 7 software (version 7.4.0.4.; Oroboros Instruments, Innsbruck, Austria) was used for online respirometry data display and analysis.

### 2.6. Plasma MPO Measurement

Circulating MPO activity, used as an indicator of systemic neutrophil activation, was measured as described by Kuebler et al. [22].

### 2.7. Statistical Analysis

Data were evaluated using the SigmaStat 13.0 software package (Systat Software, San Jose, CA, USA). The Shapiro–Wilk test was performed to analyze the normality of the data distribution. The Kruskal–Wallis analysis of variance (ANOVA) of the ranks, with Dunn’s post-hoc test, or two-way repeated measures ANOVA completed with Holm–Sidak’s post-hoc test, was employed for calculating the differences between the groups. The median values with 25th and 75th percentiles are provided in the figures; *p* < 0.05 was considered statistically significant.

## 3. Results

### 3.1. Changes in Well-Being

Animal well-being markedly declined at 6 h post-sepsis induction across all the septic groups (Supplementary Material, Supplementary Figure S1), as indicated by increasing RSS score, which continued to increase during the later stages of sepsis. Animals that reached predefined humane endpoints were euthanized at 16 h, and their RSS data were excluded from the statistical analysis. Specifically, two animals per septic group were euthanized, except in the 3–6 h CH_4_-treated group, wherein no animals met the euthanasia criteria. Considering these circumstances, untreated septic animals demonstrated the highest RSS at 16 h, whereas animals that received CH_4_ treatment at 3–6 h exhibited significantly lower scores than untreated septic controls.

### 3.2. Organ Function Alterations

**Figure 2:**
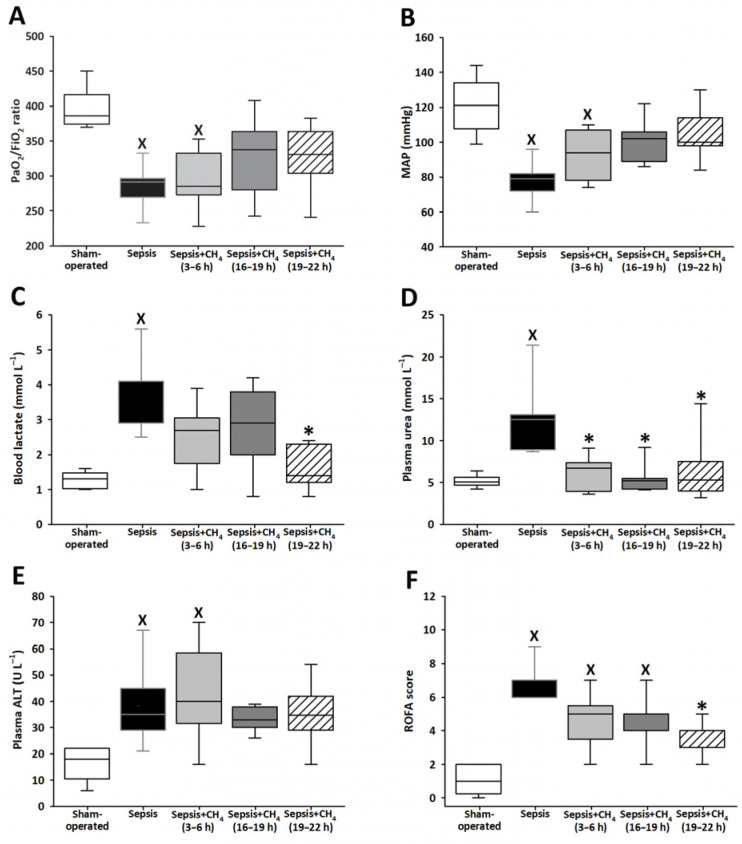
PaO_2_/FiO_2_ ratio (**A**), mean arterial pressure (MAP) (**B**), blood lactate (**C**), plasma urea (**D**), plasma alanine aminotransferase (ALT) (**E**), and Rat-specific Organ Failure Assessment (ROFA) scores (**F**) in the sham-operated group and in various groups of septic animals (untreated or treated with CH_4_ at t = 3–6 h, 16–19 h, or 19–22 h). Median with 25th and 75th percentiles; ^X^ *p* < 0.05 versus sham-operated group; * *p* < 0.05 versus untreated septic group.

Various ROFA score components were determined at t = 24 h. The untreated septic group exhibited significantly lower PaO_2_/FiO_2_ ratios and MAP values than the sham-operated controls, indicating that sepsis-induced marked respiratory dysfunction and systemic hypotension (Figure 2A,B). Septic animals that received CH_4_ treatment at t = 3–6 h demonstrated comparable reductions in these parameters. In contrast, PaO_2_/FiO_2_ ratios and MAP values in the groups receiving CH_4_ treatment at t = 16–19 and 19–22 h did not significantly differ from those of the sham-operated group.

Compared with sham-operated animals, only the untreated septic group showed elevated blood lactate and plasma urea levels. Compared with untreated sepsis, CH_4_ treatment administered in the latest stage significantly decreased the lactate levels (Figure 2C), whereas CH_4_ treatment administered during any of the three stages led to significantly decreased plasma urea levels (Figure 2D). The untreated septic group and the group receiving early CH_4_ treatment demonstrated significantly elevated plasma ALT levels, whereas ALT levels following CH_4_ administration at 16–19 and 19–22 h were comparable to those observed in the sham-operated group (Figure 2E). All the septic groups exhibited significantly elevated ROFA scores, which reflect the aggregate of systemic organ dysfunction parameters, compared with the sham-operated controls. Notably, CH_4_ treatment administered at the latest timepoint (t = 19–22 h) significantly reduced the ROFA scores compared with the untreated septic group (Figure 2F).

### 3.3. Oxygen Dynamics Alterations

**Figure 3:**
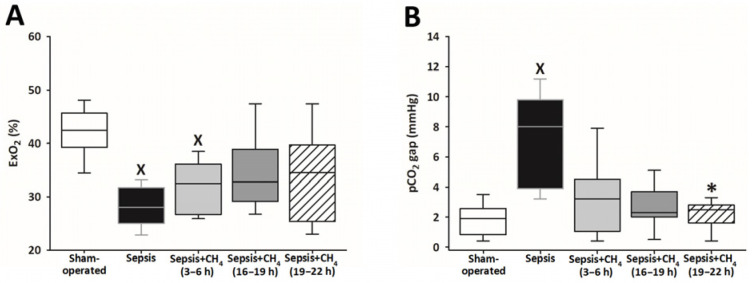
Oxygen extraction (ExO_2_) (**A**) and partial carbon dioxide gap (pCO_2_ gap) (**B**) in the sham-operated group and in various groups of septic animals (untreated or treated with CH_4_ at t = 3–6 h, 16–19 h, or 19–22 h). Median with 25th and 75th percentiles; ^X^ *p* < 0.05 versus sham-operated group; * *p* < 0.05 versus untreated septic group.

Systemic ExO_2_ and the pCO_2_ gap were measured to evaluate sepsis-induced alterations in oxygen dynamics and tissue hypoperfusion (Supplementary Material). The untreated septic group and the early CH_4_-treated group (t = 3–6 h) showed significantly lower ExO_2_ values than the sham-operated group, whereas the groups receiving CH_4_ treatment at later timepoints (t = 16–19 and 19–22 h) exhibited no significant differences (Figure 3A). Sepsis resulted in a significantly increased pCO_2_ gap solely in the untreated septic animals compared with that in the sham-operated controls, whereas the CH_4_-treated septic groups exhibited no such elevations. Furthermore, CH_4_ administration at the latest timepoint significantly decreased the pCO_2_ gap compared with the untreated septic group (Figure 3B).

### 3.4. Cerebellar and Renal Mitochondrial Function Alterations

**Figure 4:**
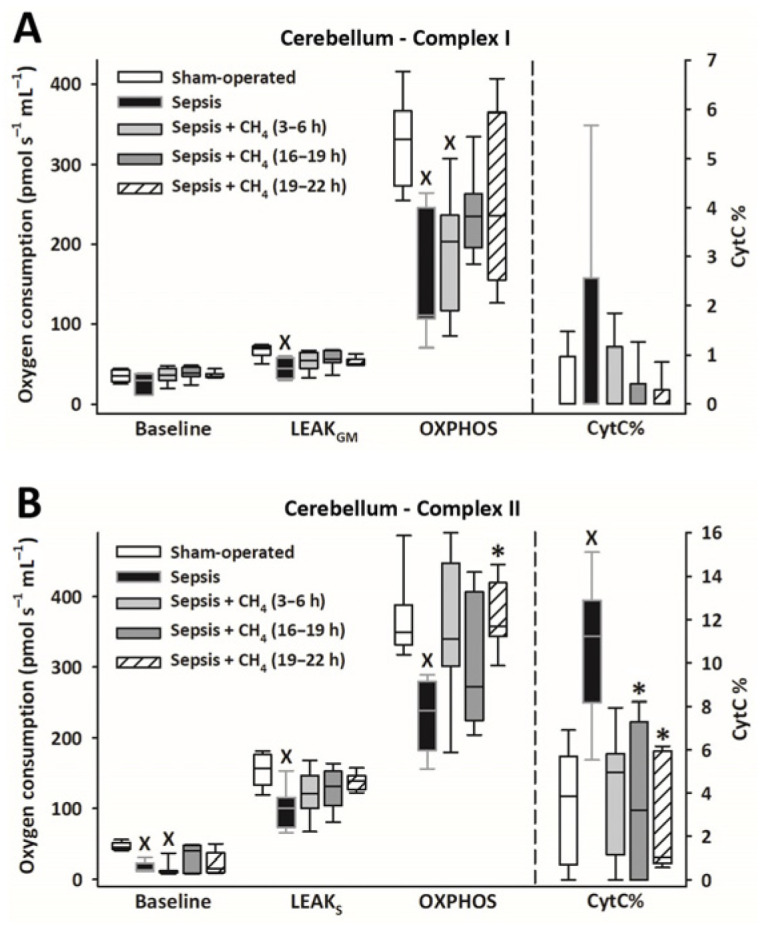
Mitochondrial respiration linked to Complex I (**A**) and II (**B**) in the cerebellum in the sham-operated group and in various groups of septic animals (untreated or treated with CH_4_ at t = 3–6 h, 16–19 h, or 19–22 h). Median with 25th and 75th percentiles; ^X^ *p* < 0.05 versus sham-operated group; * *p* < 0.05 versus untreated septic group. Respiratory states: Baseline: respiration without external substrates and inhibitors; LEAK_GM_: glutamate-malate-supported respiration; LEAK_S_: succinate-supported respiration capacity; OXPHOS: ADP-stimulated respiration capacity; CytC%: increase in oxygen consumption after addition of CytC, expressed as a percentage of OXPHOS respiration.

**Figure 5:**
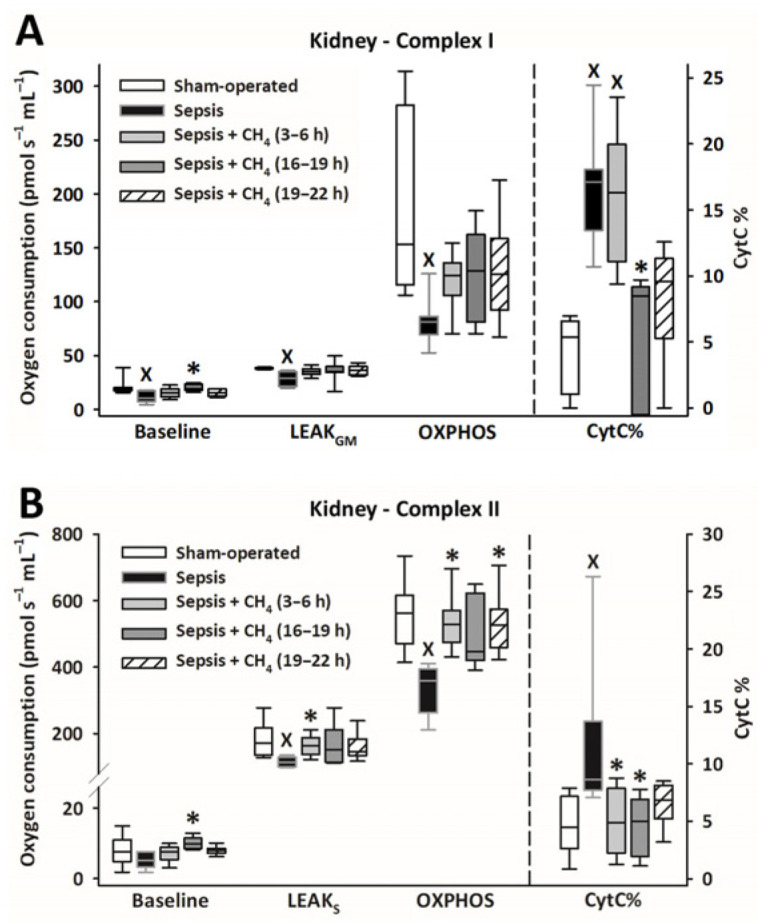
Mitochondrial respiration linked to Complex I (**A**) and II (**B**) in the kidney in the sham-operated group and in various groups of septic animals (untreated or treated with CH_4_ at t = 3–6 h, 16–19 h, or 19–22 h). Median with 25th and 75th percentiles; ^X^ *p* < 0.05 versus sham-operated group; * *p* < 0.05 versus untreated septic group. Respiratory states: Baseline: respiration without external substrates and inhibitors; LEAK_GM_: glutamate-malate-supported respiration; LEAK_S_: succinate-supported respiration capacity; OXPHOS: ADP-stimulated respiration capacity; CytC%: increase in oxygen consumption after addition of CytC, expressed as a percentage of OXPHOS respiration.

Owing to sepsis, mitochondrial respiration was markedly reduced in the cerebellum (Figure 4) and kidney (Figure 5), as evidenced by significantly lower LEAK_GM_, LEAK_S_, and OXPHOS values than those in the sham-operated group.

Compared with the untreated septic group, CH_4_ treatment did not significantly improve C-I–linked respiration in the cerebellum and kidney. However, the C-II–linked OXPHOS values significantly improved in the cerebellum following the latest (t = 19–22 h) CH_4_ treatment (Figure 4B). In the kidney, significant improvements in C-II–linked OXPHOS were observed following early (t = 3–6 h) and late (t = 19–22 h) CH_4_ treatments (Figure 5B). Mitochondrial oxygen consumption increased following the addition of cytochrome c (CytC; expressed as a proportion of OXPHOS: CytC%), indicating significant deterioration of the outer mitochondrial membrane permeability in both organs in the untreated septic group. This increase was apparent for C-II in the cerebellum (Figure 4B) and both C-I and -II in the kidney (Figure 5B). In the cerebellum, CytC% values for C-II were comparable to those of the sham-operated group following CH_4_ treatment applied at all stages of sepsis, and CH_4_ administered at t = 16–19 and t = 19–22 h showed significantly lower C-II–related CytC% values than untreated sepsis. Similarly, a time-dependent protective effect of CH_4_ on the outer mitochondrial membrane integrity was observed in the kidney for C-I (Figure 5A), whereas CH_4_ treatment at all stages conferred a beneficial effect on C-II (Figure 5B).

### 3.5. Neutrophil Granulocyte Activation

**Figure 6:**
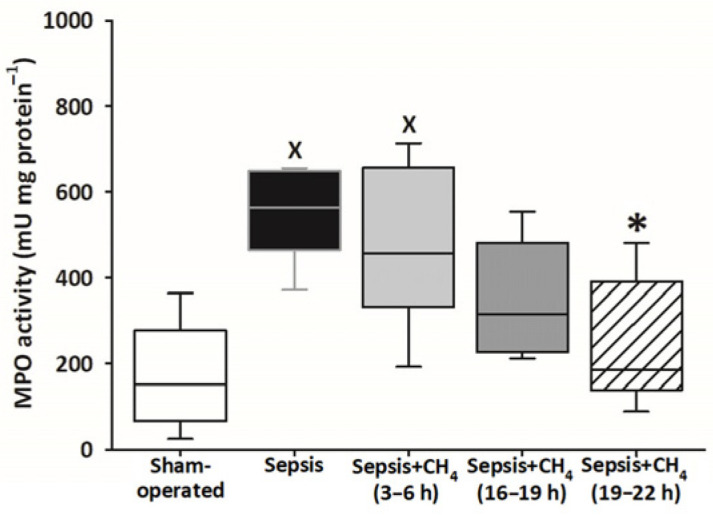
Plasma myeloperoxidase (MPO) activity in the sham-operated group and in various groups of septic animals (untreated or treated with CH_4_ at t = 3–6 h, 16–19 h, or 19–22 h). Median with 25th and 75th percentiles; ^X^ *p* < 0.05 versus sham-operated group; * *p* < 0.05 versus untreated septic group.

Compared with sham-operated animals, MPO activity in the plasma was significantly elevated only in the untreated septic group and the group receiving CH_4_ treatment at t = 3–6 h. The latest CH_4_ treatment (t = 19–22 h) resulted in a significantly lower MPO activity compared with the untreated septic group (Figure 6).

## 4. Discussion

This study demonstrated the efficacy of CH_4_ inhalation therapy in mitigating organ dysfunction, neutrophil activation, and mitochondrial respiratory impairment at various stages of a standardized intra-abdominal sepsis model [19]. To evaluate the influence of intervention timing on therapeutic outcomes, CH_4_ was administered at distinct timepoints following sepsis induction. Early (3–6 h) administration prevented sepsis-related mortality, whereas treatments initiated at later stages improved MAP; enhanced respiratory, hepatic, and renal functions, as evidenced by enhanced PaO_2_/FiO_2_ ratios and reduced urea and ALT levels; and attenuated metabolic acidosis, as indicated by decreased lactate levels.

Notably, late-stage CH_4_ treatment significantly reduced multiple organ dysfunction, as evidenced by lower ROFA scores, and yielded the greatest improvements in ExO_2_ and reductions in the pCO_2_ gap, with some benefits observed across all treated groups. These findings suggest that the attenuation of tissue hypoxia and enhancement of ExO_2_ can originate from improved DO_2_ and microcirculatory function, which is consistent with previous studies [23].

The potential transient benefits of early CH_4_ treatment remain uncertain because organ function parameters were only assessed at the endpoint. Nevertheless, our results suggest that early administration cannot significantly impact organ function during the initial and relatively preserved stage of sepsis and that its benefits can be short-lived. In contrast, CH_4_ inhalation was most effective when applied following organ dysfunction onset, supporting the concept of a time-dependent therapeutic window.

In this study, the exact mechanisms underlying the benefits of CH_4_ inhalation could not be fully understood. However, improved mitochondrial functions may play a key role (see later sections). Moreover, the anti-inflammatory properties of CH_4_ are probable contributors; however, comprehensive cytokine profiling was not performed, and only MPO activity was employed for assessing neutrophil activation. However, previous studies have demonstrated that CH_4_ suppresses proinflammatory cytokine production and inhibits NF-κB activation [17,24,25].

It should be noted that conventional sepsis therapies remain of limited effectiveness. In sepsis, alternative treatments have emerged, such as methylene blue, which improved mean arterial pressure, increased oxygenation and reduced mortality in adult patients with septic shock [26] or high-dose ascorbic acid, which has been shown to have antioxidant and anti-inflammatory effects in sepsis [27,28]. CH_4_ shows significant antioxidant activity, as indicated by the reduced activation of xanthine oxidoreductase during ischemia reperfusion [23,29]. Accordingly, CH_4_, a gaseous molecule, may broaden the scope of alternative interventions in the treatment of sepsis.

Furthermore, CH_4_ administration decreases the tissue levels of nitric oxide and nitrotyrosine [23], both of which can impair mitochondrial respiration by competing with oxygen or modifying the respiratory chain components via nitrosylation and oxidation [30]. Additionally, CH_4_ inhalation has been reported to improve organ function in an Nrf2-dependent manner during early reperfusion [31].

Despite its chemical inertness, CH_4_ exerts substantial biological activity, probably because of its apolar and hydrophobic nature, which facilitates accumulation at membrane interfaces and may modulate transmembrane proteins, ion channels, and enzyme activity [29]. Additionally, hydrocarbon gases, including CH_4_, have been demonstrated to influence membrane dynamics, affect cell–cell junction integrity, and modulate erythrocyte deformability and aggregation, particularly under oxidative stress conditions [32,33].

Sepsis-associated encephalopathy (SAE), a life-threatening complication of systemic infection, is characterized by a complex interplay of oxidative and nitrosative stress, neuroinflammation, and blood–brain barrier disruption. These pathophysiological changes ultimately result in neuronal cell death, impaired neurotransmission, and mitochondrial dysfunction within the neurons [6,34]. Mitochondrial injury, primarily induced by oxidative stress, promotes CytC release and mitochondrial permeability transition pore opening, thereby triggering apoptosis and neuronal loss [35]. In our study, CH_4_ inhalation preserved mitochondrial membrane integrity and enhanced oxidative phosphorylation efficiency, resulting in improved cellular energetics and organ function in the treated animals. The 19–22 h treated group exhibited the most pronounced benefits, including improved cerebellar mitochondrial respiration, outer mitochondrial membrane permeability stabilization, and enhanced ATP-generating capacity. These findings are consistent with those of earlier studies demonstrating that normoxic CH_4_ exposure attenuates CytC release and preserves mitochondrial respiration [14]. Complementary evidence from ischemia–reperfusion models shows that the administration of CH_4_-enriched saline not only reduces neuronal apoptosis by suppressing CytC release [36] but also mitigates hippocampal microglial activation, oxidative stress, and behavioral deficits in murine models of SAE [25].

Sepsis-induced microcirculatory dysfunction also contributes to impaired renal DO_2_ and nutrient supply, predisposing renal tubular epithelial cells, which are among the most metabolically active cells in the body, to mitochondrial damage and cellular injury [8,37,38]. The degree of mitochondrial dysfunction, characterized by impaired oxidative phosphorylation and increased uncoupling, is strongly associated with sepsis-induced AKI severity [39]. Methane therapy has previously been demonstrated to exert renoprotective effects by attenuating oxidative stress, enhancing mitochondrial respiration, and modulating endoplasmic reticulum stress, thereby restoring energy homeostasis in renal cells [16]. In the present study, CH_4_ inhalation across all treatment stages significantly decreased serum urea levels, reduced CytC release, and normalized C-II–linked oxidative phosphorylation in kidney tissues. These findings support the hypothesis that CH_4_ preserves mitochondrial membrane integrity, possibly by stabilizing the outer membrane, thereby preventing apoptosis and maintaining renal function during sepsis.

Methane therapy administered during the early, intermediate, and late stages of sepsis exerted organ-specific protective effects on mitochondrial function in the brain and kidneys. In the kidneys, all treatment windows exhibited beneficial effects, whereas in the cerebellum, efficacy was restricted to late-stage interventions. Notably, each treatment window conferred the following distinct advantages: early CH_4_ administration (3–6 h) was associated with complete survival, whereas the intermediate (16–19 h) and late (19–22 h) groups demonstrated extensive mitochondrial function enhancements.

Collectively, these findings suggest that prolonged or repeated inhaled CH_4_ administration following sepsis diagnosis can represent the most rational and effective therapeutic strategy. Our recent study further supports this concept, demonstrating that 24-h continuous CH_4_ inhalation during veno–venous extracorporeal membrane oxygenation not only significantly increased renal blood flow and urine output but also reduced proinflammatory cytokine levels in swine [18]. These results suggest that prolonged inhalation of CH_4_, initiated at an earlier stage of sepsis, could provide even more pronounced organ protection.

Some limitations of our sepsis model should be acknowledged. First, although the polymicrobial rat model used in this study provided valuable mechanistic insights, species-specific differences in the immune response, metabolism, and disease progression may have limited the direct clinical translation of these findings. Notably, broad-spectrum antibiotics, including standard first-line agents in sepsis management, were intentionally excluded from our model, as a previous study suggests that they can adversely influence mitochondrial function [40]. Second, the 24-h timeframe of this model did not enable pathogen identification and targeted antimicrobial therapy, which are integral to clinical sepsis management. The potential synergistic effects of CH_4_ treatment when combined with conventional treatments, including antibiotics and immunomodulatory agents, should be explored in future studies.

## 5. Conclusions

This study demonstrates that inhaled CH_4_ treatment, administered at different stages of early sepsis, effectively mitigates sepsis-induced organ dysfunction, mitochondrial damage, and inflammatory responses. Notably, CH_4_ treatment during the advanced stage of multiple organ dysfunction (19–22 h post-induction) was the most effective, as evidenced by improved organ function and enhanced mitochondrial performance in the cerebellum and kidneys, mainly by preserving mitochondrial oxygen consumption, particularly via C-II–linked respiration. Furthermore, CH_4_ treatment improved oxygen dynamics, with the intervention timing strongly influencing treatment efficacy.

Overall, these findings underscore the therapeutic potential of CH_4_ in sepsis management. However, to enhance translational relevance, future studies should integrate standard clinical interventions, including antibiotics and immunomodulatory agents, to assess potential synergistic effects and further optimize treatment approaches.

## Data Availability

The data used to generate the figures in the manuscript are fully included in the Supplementary Excel file. These cover all quantitative results presented. Additional raw data—such as outputs from specialized instruments (Oxygraph-2k (O2k, OROBOROS INSTRUMENTS, Austria, etc.)—are stored in proprietary formats that require specific software and expert interpretation. Therefore, these raw datasets are available upon reasonable request.

## Supplementary Materials

The following supporting information can be downloaded at: https://www.mdpi.com/article/10.3390/antiox14070814/s1, Table S1. Threshold values for components of the Rat-specific Organ Failure Assessment scoring system; Figure S1. Time course of the Rat-Specific Sickness (RSS) score in sham-operated and in various groups of septic animals (untreated or treated with CH_4_ at t = 3–6 h, 16–19 h, or 19–22 h). Median with 25th and 75th percentiles; ^X^ *p* < 0.05 versus sham-operated group; * *p* < 0.05 versus untreated septic group.

## Abbreviations

AKI	Acute Kidney Injury
ALT	Alanine Aminotransferase
ARRIVE	Animal Research: Reporting of In Vivo Experiments
ATP	Adenosine Triphosphate
C-I, C-II	Mitochondrial Complex I and II
CH_4_	Methane
CytC%	Cytochrome c–induced oxygen consumption as a percentage of OXPHOS
DO_2_	Oxygen Delivery
ECMO	Extracorporeal Membrane Oxygenation
ExO_2_	Oxygen Extraction
FiO_2_	Fraction of Inspired Oxygen
LEAKGM	Glutamate-Malate–supported Leak Respiration
LEAKS	Succinate-supported Leak Respiration
MAP	Mean Arterial Pressure
MQTiPSS	Minimum Quality Threshold in Preclinical Sepsis Studies
MPO	Myeloperoxidase
NF-κB	Nuclear Factor kappa-light-chain-enhancer of activated B cells
OXPHOS	ADP-Stimulated Oxidative Phosphorylation
PaO_2_	Partial Pressure of Arterial Oxygen
PaO_2_/FiO_2_	Arterial oxygen partial pressure/fraction of inspired oxygen
pCO_2_-gap	Central Venous-to-Arterial Carbon Dioxide Gap
ROFA	Rat-Specific Organ Failure Assessment
ROS	Reactive Oxygen Species
RSS	Rat-Specific Sickness Score
SOFA	Sequential Organ Failure Assessment
VO_2_	Oxygen Consumption
