# Supplementary material for: Time Matters: Methane Inhalation Mitigates Mitochondrial and Organ Dysfunction in Advanced Experimental Sepsis
Source: Antioxidants (Basel). 2025 Jul 1;14(7):814. doi: 10.3390/antiox14070814 (PMC12291870; doi:10.3390/antiox14070814)
Supplement: Supplementary file 1 [file antioxidants-14-00814-s001.zip › Gul燾si_et_al_SUPPLEMENTARY MATERIAL after proofreading.pdf]

SUPPLEMENTARY MATERIAL

Time Matters: Methane Inhalation Mitigates Mitochondrial and Organ Dysfunction in Advanced Experimental Sepsis

Table of contents

I. Supplementary Table S1 2

II. Supplementary Figure S1 3

I. Supplementary Table S1

TABLE S1. Threshold values for components of the Rat-specific Organ Failure Assessment scoring system

Parameters	ROFA score values				
	**0**	**1**	**2**	**3**	**4**
**Respiratory system**<br>PaO_2/FiO_2 ratio	>400	400−300	300−200	200−100	<100
**Cardiovascular system**<br>MAP (mmHg)	>75	75−65	65–55	<55	-
**Metabolism**<br>blood lactate (mmol/L)	<1.64	1.64−3	3–4	4−5	>5
**Liver function**<br>plasma ALT (U/L)	<17.5	17.5−30.2	>30.2	-	-
**Kidney function**<br>plasma urea (mmol/L)	<7.5	7.5−21	>21	-	-

ROFA: Rat-specific Organ Failure Assessment; MAP: mean arterial pressure; ALT: alanine aminotransferase

II. Supplementary Figure S1

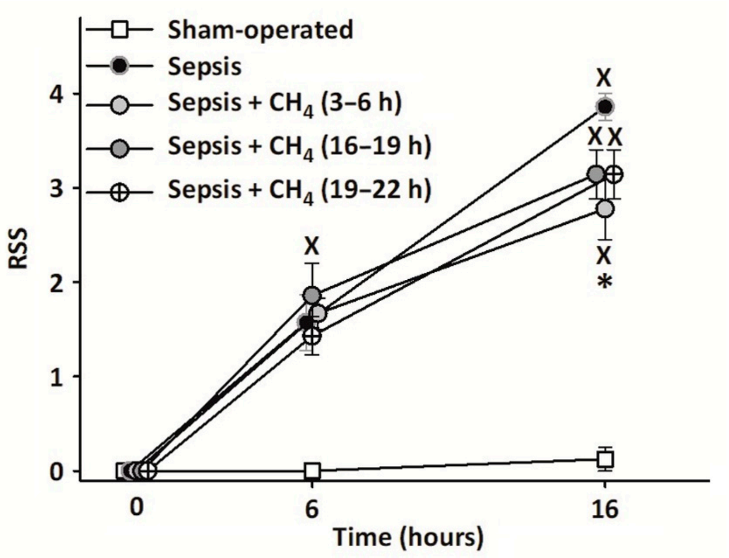

Figure S1. Time course of the Rat-Specific Sickness (RSS) score in sham-operated and in various groups of septic animals (untreated or treated with CH_4 at t = 3–6 hours, 16–19 hours, or 19–22 hours). Median with 25th and 75th percentiles; $^{X}p<0.05$ versus sham-operated group; *$p<0.05$ versus untreated septic group.
